# Supplementary material for: Comparison of Culture- and Quantitative PCR-Based Indicators of Antibiotic Resistance in Wastewater, Recycled Water, and Tap Water
Source: Int J Environ Res Public Health. 2019 Oct 30;16(21):4217. doi: 10.3390/ijerph16214217 (PMC6862664; doi:10.3390/ijerph16214217)
Supplement: Supplementary file 1 [file ijerph-16-04217-s001.pdf]

Supplementary Materials:
Table S1 - Water systems sampled and sampling dates.

Country	Water system	Samples	Dates
**Portugal (PT)**	WWTP	RWW 11	13.06.2017
		sTWW 11	
		RWW 14	26.07.2017
		sTWW 14	
**United States (US)**	Simulated reclaimed water distribution system	SRWDS 1	05.09.2017
		SRWDS 2	13.09.2017
		SRWDS 3	18.09.2017
	River	River 1	13.09.2017
		River 2	15.09.2017
		River 3	19.09.2017
	Tap water with GAC system	TW 1	05.09.2017
		TW 2	14.09.2017
		TW 3	18.09.2017
	WWTP	RWW 1	13.09.2017
		sTWW 1	
		tTWW 1	
		RWW 2	15.09.2017
		sTWW 2	
		tTWW 2	
		RWW 3	19.09.2017
		sTWW 3	
		tTWW 3	

Table S2- Quantitative-PCR conditions used in this study. US refers to protocols used in the United States and PT to protocols used in Portugal.

Target Gene	Primers	Primers sequence	Conditions	Limit of quantification (no. of copies)	Primers Reference
**16S rRNA**	1369 FW<br>1492 RV	CGGTGAATACGTTCYCGG<br>GGWTACCTTGTTACGACTT	**US:** 98°C 2 min (1 cycle), 98°C 5 s - 55°C 5 s (40 cycles)<br>Other: 3b	100	[56]
**16S rRNA**	1114F<br>1275R	CGGCAACGAGCGCAACCC<br>CCATTGTAGCACGTGTGTAGCC	**PT:** 95°C for 10 min (1 cycle); 95°C for 15 s, 55°C for 20 s and 72°C for 10 s (35 cycles)<br>Other: 1a	402	[57]
**23S rRNA**	entero FW<br>entero RV<br>Probe	AGAAATTCCAAACGAACTTG<br>CAGTGCTCTACCTCCATCATT<br>TGGTTCTCTCCGAAATAGCTTTAGGGCTA	**US:** 50°C 2 min (1 cycle), 95°C 10 min - 95°C 15 s - 60°C 60 s (40 cycles)<br>Other: 4a	1	[35]
gadAB	gadrt-1<br>gadrt-2	GCGTTGCGTAAATATGGTTGCCGA<br>CGTCACAGGCTTCAATCATGCGTT	**US**: 95°C 2 min (1 cycle), 95°C 5 s - 69°C 5 s (40 cycles)<br>Other: 3b / **PT**: 95°C 10 min (1 cycle), 95°C 15 s - 60°C 1 min (40 cycles)<br>Other: 1b	**US**: 10 / **PT**: 43	[36]
sul1	sul1 FW<br>sul1 RV	CGCACCGGAAACATCGCTGCAC<br>TGAAGTTCCGCCGCAAGGCTCG	**US**: 98°C 2 min (1 cycle), 98°C 5 s - 69°C 5 s (40 cycles)<br>Other: 3b / **PT**: 95°C 5 min (1 cycle), 95°C 10 s - 60°C 30 s (35 cycles)<br>Other: 5d	**US**: 100 / **PT**: 96	[40]
sul2	sul2 FW<br>sul2 RV	TCCGGTGGAGGCCGGTATCTGG<br>CGGGAATGCCATCTGCCTTGAG	**US**: 98°C 2 min (1 cycle), 98°C 5 s - 67.5°C 5 s (40 cycles)<br>Other: 3b / **PT**: 95°C 5 min (1 cycle), 95°C 15 s - 60°C 1 min (40 cycles)<br>Other: 1a	**US**: 100 / **PT**: 47	[40]
***tet*(A)**	tetA FW<br>tetA RV	GTAATTCTGAGCACTGTCGC<br>CATAGATCGCCGTGAAGAGG	**US**: 98°C 2 min (1 cycle), 98°C 5 s - 57.2°C 10 s - 72°C 15 s (40 cycles)<br>Other: 3b / **PT**: 95°C 10 min (1 cycle), 95°C 15 s -	**US**: 100 / **PT**: 51	[43]

			60°C 1 min (40 cycles)<br>Other: 1c		
***tet*(O)**	tetO FW<br>tetO RV	ACGGARAGTTTATTGTATACC<br>TGGCGTATCTATAATGTTGAC	**US:** 98°C 2 min (1 cycle), 98°C 5 s - 50.3°C 5 s (40 cycles)<br>Other: 3b / **PT**: 95°C 10 min (1 cycle), 95°C 15 s - 60°C 1 min (40 cycles)<br>Other: 1a	**US**: 100 / **PT**: 41	[42]
bla$_{OXA-1}$	oxa1 FW<br>oxa1 RV	TATCTACAGCAGCGCCAGTG<br>CGCATCAAATGCCATAAGTG	**US**: 98°C 2 min (1 cycle), 98°C 5 s - 62°C 5 s (40 cycles)<br>Other: 3b / **PT**: 95°C 10 min (1 cycle), 95°C 15 s - 60°C 1 min (40 cycles)<br>Other: 2a	**US**: 10 / **PT**: 38	[38]
***uid*A**	uidA-FW<br>uidA-RV	CAACGAACTGAACTGGCAGA<br>CATTACGCTGCGATGGAT	**PT:** 95°C 10 min (1 cycle), 95°C 15 s - 60°C 1 min (40 cycles)<br>Other: 2a	79	[37]
bla$_{CTX-M}$	CTXM-FW<br>CTXM-RV	CTATGGCACCACCAACGATA<br>ACGGCTTTCTGCCTTAGGTT	**PT:** 95°C 10 min (1 cycle), 95°C 15 s - 60°C 1 min (40 cycles)<br>Other: 2a	29	[39]
***intI*1**	intI1-LC1<br>intI1-LC5	GCCTTGATGTTACCCGAGAG<br>GATCGGTCGAATGCGTGT	**PT:** 95°C 10 min (1 cycle), 95°C 15 s - 60°C 1 min (40 cycles)<br>Other: 2a	54	[38]

1) KAPA SYBR® FAST ABI Prism® qPCR Master Mix; 2) SYBR® Select Master Mix; 3) EvaGreen Supermix; 4) Probes Supermix; 5) FAST SYBR; a) 200 nM of primer; b) 400 nM of primer; c) 600 nM of primer; d) 300nM of primer.

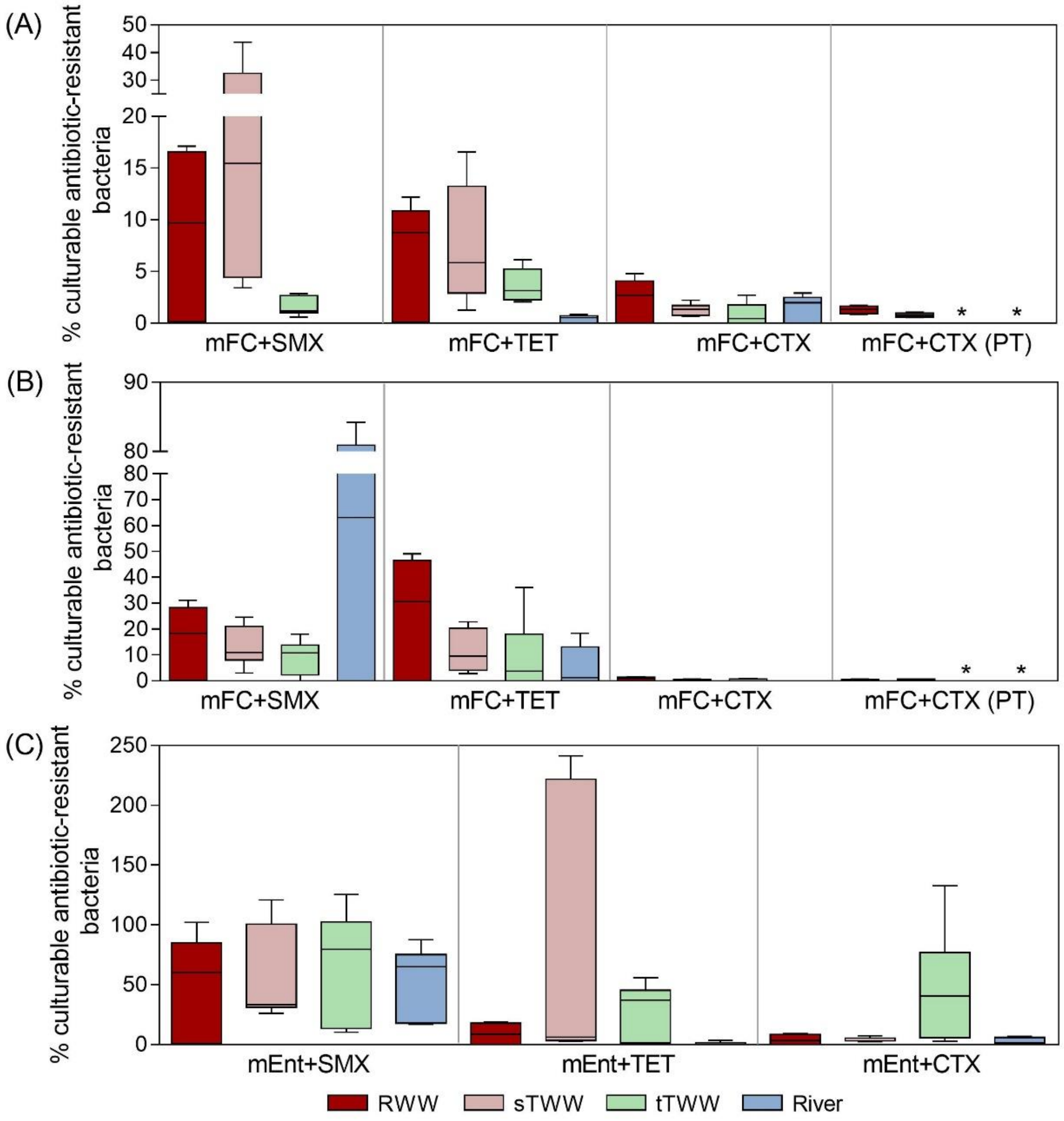

Figure S1 – Percentage of "resistant" bacteria (i.e., growing in presence of target antibiotic at select concentration): (A) presumptive total coliforms, (B) presumptive faecal coliforms and (C) presumptive enterococci in the respective samples, defined as colony forming units (CFU) on cultures with antibiotics relative to CFU on cultures without antibiotics. RWW: wastewater treatment plant influent; sTWW: wastewater collected after secondary wastewater treatment; tTWW: wastewater collected after UV disinfection wastewater treatment. PT refers to data obtained of samples collected in Portugal, the other data presented refer to data obtained of samples collected in United States. * - not determined. SMX: sulfamethoxazole (350 mg/L); CTX: cefotaxime (4 mg/L); TET: tetracycline (16 mg/L).

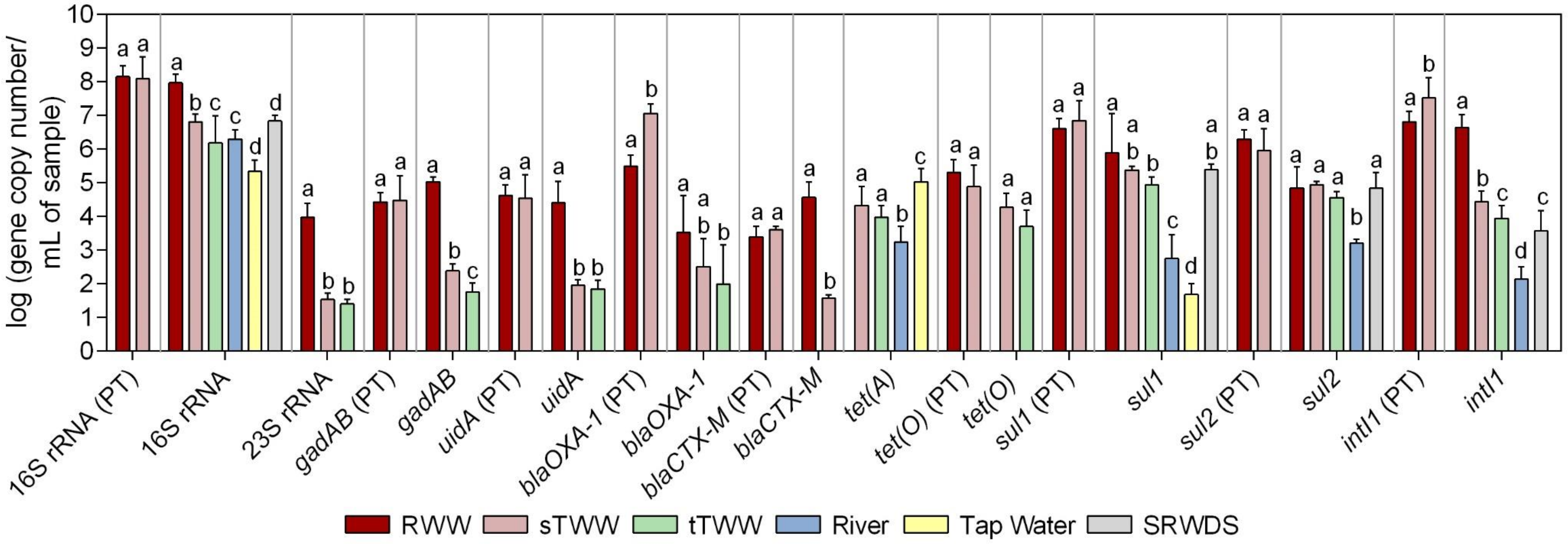

Figure S2 – Absolute abundance values (gene copy number/mL of sample) of the target genes in the six water types: RWW: wastewater treatment plant influent; sTWW: wastewater collected after secondary wastewater treatment; tTWW: wastewater collected after UV disinfection wastewater treatment; River; Tap water and SRWDS: *simulated* reclaimed water distribution system. PT refers to data obtained of samples collected in Portugal, the other data presented refer to data obtained of samples collected in United States. Error bars represent the standard deviation (n=3 and n=2 independent samples, for US and PT, respectively). In PT samples, the genes 23S rRNA and *tet*(A) were not analysed. In US, the absence of the genes *bla*$_{OXA-1}$, *tet*(A), *tet*(O), *sul2* and *intI*1 is due to their quantification below the limit of detection while the absence of the genes 23S rRNA, *gadAB*, *uidA* and *bla*$_{CTX-M}$ was is due to genes quantification below the limit of quantification. a, b, c, and d indicate significantly ($p<0.01$) different groups comparing the different types of water.
